# The protective effect of crocin on cisplatin-induced testicular impairment in rats

**DOI:** 10.1186/s12894-021-00889-2

**Published:** 2021-09-01

**Authors:** Behzad Mesbahzadeh, Mohammadmehdi Hassanzadeh-Taheri, Mohadese-sadat Aliparast, Pardis Baniasadi, Mehran Hosseini

**Affiliations:** 1 Department of Physiology, School of Allied Medical Sciences, Birjand University of Medical Sciences, Birjand, Iran; 2 Cellular and Molecular Research Center, Birjand University of Medical Sciences, Birjand, Iran; 3 Student Research Committee, Birjand University of Medical Sciences, Birjand, Iran; 4 Present Address: Department of Anatomical Sciences, Faculty of Medicine, Birjand University of Medical Sciences, Birjand, Iran

**Keywords:** Cisplatin, Crocin, Germinal epithelium, Lipid peroxidation, Testis

## Abstract

**Background:**

Side effects of cisplatin (CIS) such as testicular toxicity restrict its clinical use. Instead, evidence indicates that crocin (CR) has synergistic anti-cancer potential with CIS and exhibited beneficial effects on CIS-induced hepatorenal damage. The aim of this study was to investigate the protective potential of CR against CIS-induced testicular toxicity in rats.

**Methods:**

Fifty adult male Wistar rats randomly assigned to five equal groups including control, CIS, and CIS plus CR at doses of 6.25 mg/kg (CIS + CR6.25), 25 mg/kg (CIS + CR25), and 100 mg/kg (CIS + CR100). CIS and CIS + CR groups received a single intraperitoneally (i.p.) injection of CIS (7 mg/kg). CR (6.25–100 mg/kg i.p.) injections were started three days before the CIS injection and continued once a day for up to 13 days. On the 14th day, all animals were sacrificed and their blood samples and testes were removed for biochemical and histological analyses.

**Results:**

Compared to the control group, CIS significantly decreased relative testis weight (0.28 vs. 0.39, *p* < 0.001), testosterone level (0.3 vs. 2.31 ng/mL, *p* < 0.001), germinal layer area (25,886 vs. 35,320 µm^2^, *p* < 0.001), superoxide dismutase (SOD) (0.9 vs.1.73 U/mg, *p* < 0.001) and increased testicular lipid peroxidation (3.05 vs. 15.35 nmol/mg, *p* < 0.001). CR at 25 mg/kg ameliorated testicular lipid peroxidation and enhanced SOD activity compared to CIS group (*p* < 0.05). Besides, CR treatment at the maximum dose (100 mg/kg) resulted in reversing CIS effects on testis weight, testosterone level, SOD, lipid peroxidation, and germinal layer area.

**Conclusions:**

These findings demonstrated that CR co-treatment could prevent CIS-induced testicular toxicity in rats.

## Background

Cisplatin (CIS) is one of the most important chemotherapeutic agents used to control several types of cancers. Despite its promising anti-cancer potential, CIS can adversely affect liver and kidney functions and induce testicular toxicity [1–5]. Nevertheless, CIS benefits outweigh its disadvantages, and currently, it is one of the most widely used chemotherapeutic agents for hematologic and solid tumor malignancies. The U.S. Food and Drug Administration approved CIS for the treatment of advanced ovarian and testicular cancers as well as bladder carcinoma [6]. However, clinical evidence demonstrates that CIS is used to treat a wide range of malignancies (off-label) [7]. Mechanistically, CIS binds to the purine bases of DNA and leads to strand breaks. Consequently, the damaged DNA, RNA, and proteins activate DNA repair mechanisms that lead to apoptotic or non-apoptotic cell death [8]. Several studies found that CIS causes severe testicular damage through impairment of Leydig cell function (inhibiting testosterone production) and induction of germ cell apoptosis. Besides the DNA damage, excessive production of reactive oxygen species (ROS) is also implicated as one of the main causes of CIS-induced testicular damage [9].

Previous studies revealed that several natural products such as eugenol, curcumin and arjunolic acid could limit CIS-induced testicular damage by reducing oxidative stress and anti-inflammatory properties [10–15]. Considering everything, natural product research is regarded as a powerful approach for developing efficient, safe, and convenient drugs [16]. Hence, there is a great interest in combining antineoplastic drugs with natural products to enhance their efficacy while minimizing side effects through the delivery of lower doses [17]. CIS can be used singly or in combination therapy for induction and neoadjuvant therapy in the clinic. Therefore, it makes CIS a suitable candidate for the combination therapy formula [18].

Crocin (CR) is the main active constituent of saffron (*Crocus sativus*) with a wide range of pharmacological properties such as antidiabetic, anti-inflammatory, antioxidant, antidegenerative, and anti-cancer [19]. Interestingly, recent studies have indicated that CR enhanced chemosensitivity of some cancerous cell lines’ to CIS. CR in combination therapy with CIS exhibited synergistic effects by induction of cell cycle arrest and apoptosis [20–22]. These findings indicate that there is no adverse interaction between CR and CIS in combination therapy. On the other hand, some works have shown that CR attenuated CIS-induced liver and kidney damage in rats [23, 24]. Therefore, it is of interest to know whether CR protective effect against CIS-induced testicular damage is still valid or not. This study aimed to investigate the potential protective effect of CR against CIS-induced testicular toxicity in rats.

## Methods

### Experimental animals

All procedures involving animals were according to the guides and rules in care and use of Laboratory Animals in Scientific Affairs with the Iranian Ministry of Health and Medical Education (2019) based on the 1964 Helsinki declaration and its later amendments or comparable ethical standards. Moreover, the animal experiments were approved by the Birjand University of Medical Sciences ethics committee (permit code: Ir.bums.REC.1398.066) in compliance with the ARRIVE guidelines [25].

Adult male albino rats (Wistar strain) weighing 180–200 g (8-week old) were used in the present study. All animals were housed in polypropylene cages in a temperature-controlled room (24 ± 2 °C) with 30–35 % relative humidity and a 12-h light/dark cycle. Besides, rats had *ad libitum* access to water and standard laboratory animal chow (Behparvar Co., IR) during the study period.

### Study design and treatments

A total of 50 rats were randomly allocated into the following five equal groups (n = 10): Control group, CIS model group (CIS), CIS plus CR at the dose of 6.25 mg/kg (CIS + Cr6.25), CIS plus CR at the dose of 25 mg/kg (CIS + CR25) and CIS plus CR at the dose of 100 mg/kg (CIS + CR100). The CR doses were selected based on previous studies revealing both protective and anti-cancer potentials. Accordingly, some studies showed that CR exhibited more anti-cancer potential at the lower doses, while others reported testicular or hepatorenal protective efficacies at higher doses [23, 24, 26]. Hence, a wide range of CR doses (6.25–100 mg/kg) were tested in this study.

The control and CIS groups only received intraperitoneal (i.p.) injections of normal saline once a day for 13 consecutive days. The Cr-treated groups (CIS + CR6.25-100) received i.p. injections of CR (Product No. 17,304, Sigma Aldrich, U.S.) dissolved in normal saline at 6.25 mg/kg, 25 mg/kg, and 100 mg/kg doses once a day for 13 consecutive days. On the 3rd day, all groups (except for control) received a single i.p. injection of CIS (PL 04515/0026, Hospira, U.K.) at the dose of 7 mg/kg. The study period and CIS dose were carefully chosen based on previous studies [1, 12, 14].

All animals were weighed on the 14th day and then sacrificed under anesthesia with ketamine-xylazine (65:10 mg/kg i.p.) [27]. Blood samples were collected by cardiac puncture for hormonal assay. Immediately after blood collection, testes were dissected out and weighed. Afterwards, the right testis was fixed in 4% paraformaldehyde solution for histology, while the left testis was kept in liquid nitrogen at − 70 °C for antioxidant assay.

### Testosterone assessment

Blood samples were centrifuged for 15 min at 3000 rpm, then sera were obtained, and testosterone assay performed using a commercially ELISA kit (Monobind, Inc., U.S.) following the manufacturer’s instruction.

### Lipid peroxidation assessment

An equal amount (100 mg) of each testis sample was homogenized in 900 µL of 0.1 M phosphate buffered saline (PBS, pH 7.4). Then, centrifuged at 4500 rpm for 15 min at 4 °C and its supernatant was collected. Total protein content was quantified using a commercial protein assay kit (NS-15,073, Navand Salamat, Iran).

Lipid peroxidation in the testicular tissue samples evaluated by the thiobarbituric acid reactive species (TBARS) method. In this method, the level of malondialdehyde (MDA), the main end product of the lipid peroxidation process, is calculated. In brief, 100 µL supernatant was added to 200 µL of 0.67% thiobarbituric acid and 600 µL of 1% O-phosphoric acid and the mixture was placed in a water bath (90 ℃ ) for 45 min. Then, the reaction was stopped by placing samples on ice and 800 µL N-butanol was added to each sample, vortexed, and the butanol phase was separated by centrifugation at 5000 rpm for 20 min. The supernatant was collected (200 µL) and its absorbance was measured spectophotometrically at 532 nm. Finally, the MDA level was expressed as nmol/mg protein [28].

### Measurement of superoxide dismutase (SOD) activity

The SOD activity in the testis homogenates were evaluated by measuring the reduction rate of the water-soluble tetrazolium WST-1 dye. The reaction mixture was prepared by adding 100 µL of 10 mM WST-1 solution, 100 µL of 2 mg/mL catalase, 5 µL of xanthine oxidase (with the final concentration of 4.5 mU/mL) and 45 µL of assay buffer (50 mM Na3PO4, 0.1 mM diethylenetriamine pentaacetic acid, and 0.1 mM hypoxanthine in 20 mL). In brief, 50 µL supernatant and 250 µL of the reaction mixture were added into each well of a 96-well plate and mixed appropriately and incubated for 5 min at room temperature. The absorbance was read at 405 nm, and the SOD activity was expressed in terms of U/mg protein [29].

### Histopathological evaluations

Tissue samples of testes were processed for paraffin-embedding by routine histological procedures, and serial Sec. (5 μm thickness) were prepared. The slides were stained with hematoxylin and eosin and analyzed under a light microscope (Euromex-CMEX-10). The quantitative parameters including seminiferous tubule area (µm^2^) and germinal layer area (µm^2^) were measured using Image J Software (1.44p; National Institute of Health, U.S.) as previously described [30, 31]. Moreover, the coefficient of germinal layer area to seminiferous tubule area was calculated.

To categorize the spermatogenesis efficacy, Johnsen’s scoring (JS) system was applied. Briefly, the degree of testicular damages was tested using a 1–10 point’s scale ranging from the lowest score of 1, indicating no seminiferous epithelium, to 10, representing complete spermatogenesis and perfect tubules (Table 1) [31]. All the evaluations were done blindly.

**Table. 1:** Johnsen score description

Johnsen score	Description
10	Complete spermatogenesis and perfect tubules
9	Slightly impaired spermatogenesis, many late spermatids, disorganized epithelium
8	Less than five spermatozoa per tubule, few late spermatozoa
7	No spermatozoa, no late spermatids, many early spermatids
6	No spermatozoa, no late spermatids, few early spermatids
5	No spermatozoa, no late spermatids, many spermatocytes
4	No spermatozoa and spermatids, few spermatocytes
3	Spermatogonia only
2	No germinal cell, Sertoli cells only
1	No seminiferous epithelium

### Statistical analysis

Data were expressed as mean ± standard deviation. Analyzes were performed using SPSS software, version 22. The homogeneity of data was checked by Shapiro–Wilk test. The differences between groups were determined with ANOVA and Dunnett’s T3 post hoc tests. Statistical significance was inferred at p < 0.05.

## Results

### Effects on body and testicular weight changes

The means of body weight, testis weight and relative testicular weight (testis weight/ body weight) of the studied groups are presented in Table 2. The CIS-treated animals exhibited lower body-weight, absolute and relative testicular weights compared to the control group (*p* < 0.001 all). Compared to the CIS group, CR treatment could significantly prevent body weight loss (*p* = 0.04), testicular weight loss *(p* < 0.001), and fall of relative testicular weight (*p* < 0.001) only at the maximum dose (100 mg/kg) in CIS-treated rats. However, these parameters were still statistically lower than normal ranges.

**Table. 2:** Effects of crocin treatment (6.25–100 mg/kg) on body weight, absolute and relative testis weights

Groups	Body weight (g)	Testis weight (g)	Relative testis weight(testis weight /body weight)*100
Control	201.62 ± 7.97	1.23 ± 0.10	0.61 ± 0.06
CIS (7 mg/kg)	187.98 ± 4.59***	0.65 ± 0.05***	0.35 ± 0.02***
CIS + CR6.25 mg/kg	186.00 ± 6.26***	0.73 ± 0.10***	0.39 ± 0.05***
CIS + CR25 mg/kg	185.38 ± 2.13***	0.70 ± 0.09***	0.38 ± 0.05***
CIS + CR100 mg/kg	195.40 ± 4.75^#^	0.94 ± 0.09**^,###^	0.48 ± 0.04***^,###^

Values are expressed as mean ± standard deviation (n = 10). CIS: cisplatin; CR: crocin; CIS + CR6.25-100: cisplatin (7 mg/kg) injected groups treated with 6.25–100 mg/kg crocin. *p < 0.05, **p < 0.01 and ***p < 0.001 in comparison with the normal control group. ^#^*p* < 0.05, ^##^*p* < 0.01 and ^###^*p* < 0.001 in comparison with the CIS group

### Effects on the serum testosterone

**Fig. 1:**
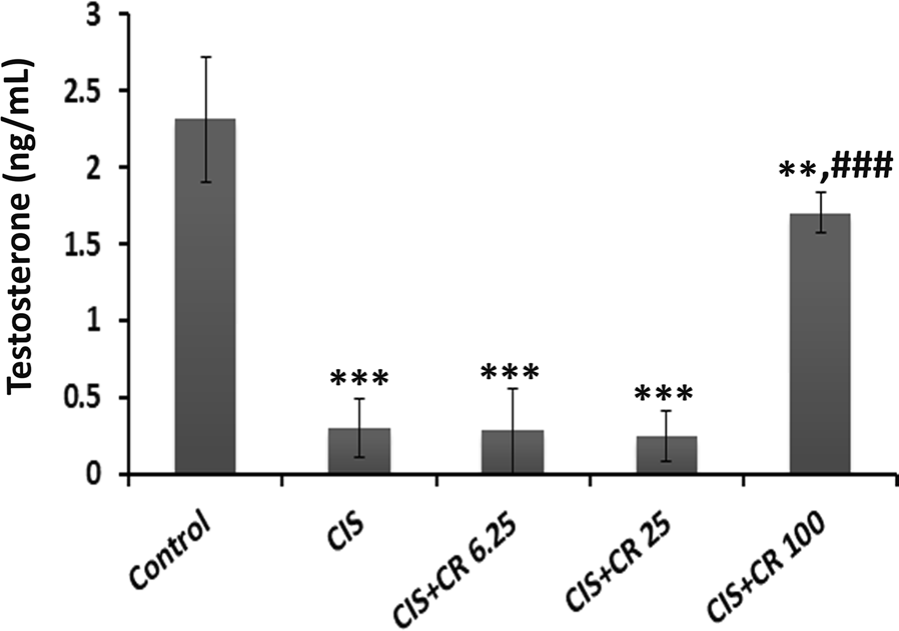
Effects of crocin treatment on plasma testosterone .Values are expressed as mean ± standard deviation (n = 10). CIS: cisplatin; CR: crocin; CIS + CR6.25-100: CIS (7 mg/kg) injected animals treated with 6.25–100 mg/kg CR. **p < 0.01 and ***p < 0.001 in comparison with the normal control group. ^###^*p* < 0.001 in comparison with the CIS group

A significant reduction (*p* < 0.001) in serum testosterone concentration was observed in the CIS-treated group compared to the control group (0.3 ± 0.19 vs. 2.31 ± 0.4 ng/mL, *p* < 0.001). CR treatment at doses up to 25 mg/kg could not prevent serum testosterone reduction in CIS-treated animals (Fig. 1). However, CR administration at the dose of 100 mg/kg caused a significant increase in serum testosterone level compared to the CIS-treated group (1.70 ± 0.13 vs. 0.3 ± 0.19 ng/mL, *p* < 0.001). Meanwhile, the testosterone level in the CIS + Cr100 group was still lower than the control group (*p* = 0.003).

### Effects on MDA and SOD

**Fig. 2:**
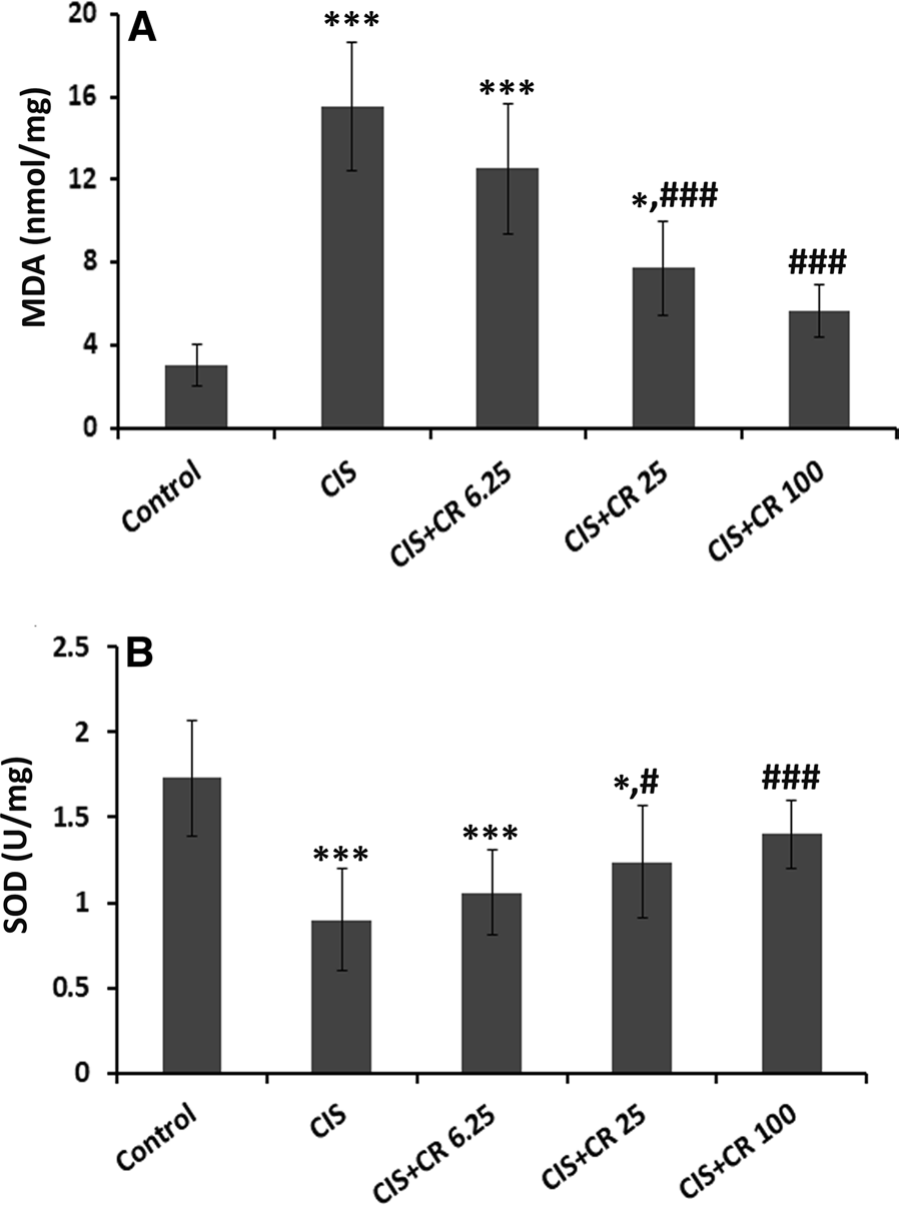
Effects of crocin treatment on testis lipid peroxidation (**A**) and superoxide dismutase (**B**). Values are expressed as mean ± standard deviation (n = 10). MDA: malondialdehyde; SOD: superoxide dismutase; CIS: cisplatin; CR: crocin; CIS + CR6.25-100: CIS (7 mg/kg) injected animals treated with 6.25–100 mg/kg CR. *p < 0.05 and ***p < 0.001 in comparison with the normal control group. ^#^*p* < 0.05, and ^###^*p* < 0.001 in comparison with the CIS group

The testicular MDA level of CIS-treated rats was statistically higher than their counterparts in the control group (15.53 ± 3.07 vs. 3.05 ± 1.05 nmol/mg, *p* < 0.001) (Fig. 2A). CR treatment at the doses of 25 mg/kg and 100 mg/kg could significantly keep MDA concentration lower than the CIS group (7.70 ± 2.29 in CIS + CR 25 mg/kg and 5.63 ± 1.27 nmol/mg protein in CIS + CR100 mg/kg). CR treatment could keep the MDA level close to the normal level only at the 100 mg/kg dose so that there was no significant difference between CIS + CR100 and control groups (*p* = 0.403).

CIS administration significantly suppressed the SOD activity in the testis tissue compared to the control group (0.9 ± 0.3 vs. 1.73 ± 0.34 U/mg, *p* < 0.001) (Fig. 2B). CR treatment at the doses of 25 mg/kg and 100 mg/kg could significantly enhance the SOD activity compared to the CIS group (*p* < 0.05 and *p* < 0.001 respectively).

### Testis histopathology

**Fig. 3:**
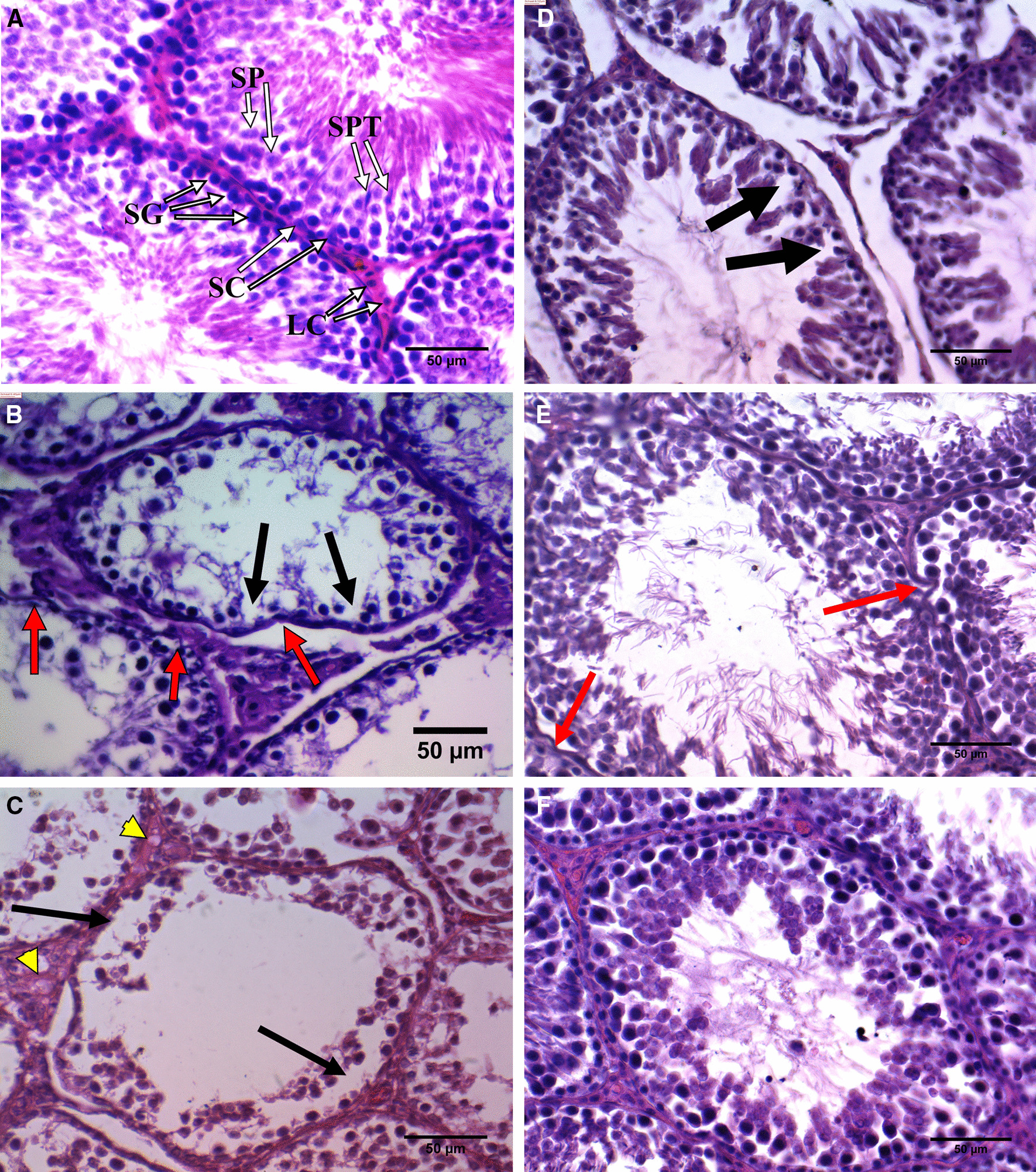
Effects of crocin treatment on the testis histology of cisplatin-treated rats. Light microscope photomicrograph of the testicular tissue of the control group (**A**) cisplatin (**B**, **C**) and cisplatin plus crocin at the doses of 6.25 mg/kg (**D**), 25 mg/kg (**E**) and 100 mg/kg (**F**). SG: spermatogonium; SP: spermatocyte; SPT: spermatid; SC: Sertoli cell; LC: leydig cell. Red arrows mark extensive shrinkages, black arrows indicate sloughing of germinal epithelium and yellow arrowheads show vacuolation. Sections were stained with Hematoxylin and eosin (400X, bars = 50 μm)

In the control group, histological examination of testis samples revealed the normal structure of seminiferous tubules in which different phases of spermatogenic cells were evident. In the germinal layer, proliferating germ cells including spermatogonia, Sertoli cells, primary spermatocytes and spermatids were radially arranged toward the lumen (Fig. 3A). However, CIS-treated rats’ seminiferous tubules showed extensive shrinkage, disruption in spermatogenesis, increased tubular lumen caused by sloughing of germinal epithelium (Fig. 3B, C). In the CIS-treated animals germinal layer area and its coefficient to seminiferous area were markedly reduced (*p* < 0.001) compared to the control group (Table 3). CR treatment could not completely restore the CIS-related damage at doses of 6.25 mg/kg (Fig. 3D) and 25 mg/kg (Fig. 3E); however, at 100 mg/kg it preserved the overall structure of seminiferous tubules close to normal condition (Fig. 3F).

**Table 3:** Effects of crocin (6.25–100 mg/kg) on the seminiferous tubule area and germinal layer area

Groups	SA (µm^2^) × 10^3^	EA area (µm^2^) × 10^3^	EA/SA * 100
Control	44.61 ± 8.10	35.32 ± 4.75	81.93 ± 8.89
CIS (7 mg/kg)	43.08 ± 2.94	25.89 ± 4.70***	59.08 ± 12.07**
CIS + CR6.25 mg/kg	42.53 ± 5.03	22.56 ± 4.18***	56.35 ± 8.19**
CIS + CR25 mg/kg	43.31 ± 5.12	23.96 ± 3.80***	59.48 ± 13.78**
CIS + CR100 mg/kg	44.83 ± 6.61	33.59 ± 6.40^#^	69.38 ± 10.98^* #^

Values are expressed as mean ± standard deviation (n = 10). CIS: cisplatin; CR: crocin; CIS + CR 6.25–100: cisplatin (7 mg/kg) injected groups treated with 6.25–100 mg/kg crocin; SA: seminiferous tubule area; EA: germinal layer area. *p < 0.05, **p < 0.01 and ***p < 0.001 in comparison with the normal control group. ^#^*p* < 0.05, ^##^*p* < 0.01 and ^###^*p* < 0.001 in comparison with the CIS group

While there was no difference between the studied groups in the seminiferous area, a significant reduction in the germinal layer area and its coefficient to seminiferous area was observed in the CIS group compared to the control group (*p* < 0.001). Treatment with CR at the maximum dose (100 mg/kg) efficiently improved the germinal epithelium in comparison with the CIS group (*p* < 0.05). The germinal layer area to seminiferous area coefficient reveals more reliable details regarding germinal layer changes. Accordingly, 81% of the seminiferous area was filled with germinal epithelium in the control group, while this rate decreased to 59% in the CIS group. This decrease is statistically significant (*p =* 0.003). Treatment with CR at doses of 6.25 mg/kg and 25 mg/kg could not inhibit germinal epithelium loss in CIS-treated animals. Administration of CR at the dose of 100 mg/kg prevented germinal epithelium disruption and kept germinal layer area around 69% of the seminiferous area, which was statistically higher than the CIS group (*p* = 0.02). Nevertheless, the germinal layer area coefficient in the CIS + CR100 group was still lower than the control group (*p* = 0.04).

**Fig. 4:**
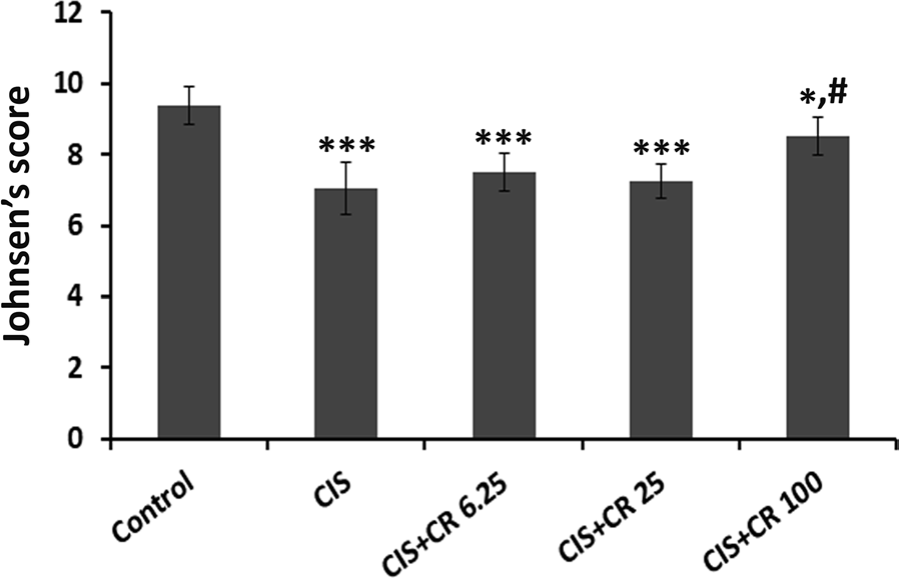
Johnsen’s scoring of testicular damage and spermatogenesis in the studied groups. Each bar indicates mean ± standard deviation. CIS: cisplatin; CR: crocin; CIS + CR6.25-100: cisplatin (7 mg/kg) injected animal treated with 6.25–100 mg/kg crocin. *p < 0.05 and ***p < 0.001 in comparison with the normal control group. ^#^*p* < 0.05 in comparison with the CIS group

The results of JS of spermatogenesis are shown in Fig. 4. The control group exhibited the maximum score (mean score = 9.7), whereas CIS-treated rats had a significantly lower score (mean score = 7.6, *p* < 0.001). CR treatment could never keep spermatogenesis at the normal level at any dose. A slight improvement was observed only in the CIS + CR100 group (mean score = 8.5), which was statistically higher than the CIS group score (mean score = 7.6, *p* = 0.03).

## Discussion

Chemotherapy is often accompanied by some side effects depending on several factors such as chemotherapy duration, dose, patient’s age, etc. [32]. Testicular toxicity is one of the most critical challenges in cancer chemotherapy leading to the emergence of a new medical discipline called oncofertility. The oncofertility aims to preserve the fertility of patients who received chemotherapy or radiotherapy. Testicular toxicity is one of the most important chemotherapy side effects restricting the use and efficacy of antineoplastic drugs, such as CIS. In the present study, the protective efficacy of CR was tested on CIS-induced testicular damage in rats. The results demonstrated that Cr, a natural product with anti-cancer efficacy, attenuated CIS-induced testicular damage in rats.

Testicular relative weight is a good indicator of normal spermatogenesis, frequently measured in reproductive studies [30]. CIS administration (7 mg/kg) significantly reduced the relative testicular weight in the current study, which is consistent with previous reports [10, 12, 33]. CR treatment only at the maximum dose (100 mg/kg) significantly prevented testicular relative weight loss in CIS-treated rats. These results go beyond previous reports, showing that CR has increased the testicular weight in nicotine/paraquat-induced mice testicular damage [34, 35].

The production of testosterone and sperms is the primary function of the testis [36]. Consequently, testosterone measurement is considered as a marker indicating optimal testicular function. In this study, CIS administration caused a severe drop in serum testosterone levels, which is in line with previous studies’ findings [13, 37]. The results showed that CR had no significant effect on testosterone reduction in CIS-treated rats at doses of 6.25–25 mg/kg, whereas it could attenuate testosterone reduction significantly at the dose of 100 mg/kg. These results are consistent with the findings reported by Salahshoor and coworkers. They have treated mice with different doses of CR (12.5, 25, and 50 mg/kg i.p.) plus nicotine (2.5 mg/kg i.p.) for four weeks. They found that nicotine administration significantly reduced testosterone in mice, while treatment with CR resulted in testosterone increase in nicotine-treated mice only at the maximum dose (50 mg/kg) [35].

Seminiferous tubules are responsible for about 80% of the total testicular volume. In the cross-section, the lumen of the seminiferous tubule is lined by Sertoli cells. It contains spermatozoa in various stages of development, from spermatogonia near the base of tubules to progressively mature forms (spermatocytes, spermatids, and spermatozoa) arranged towards the center of the lumen [36]. The most common pathological feature observed concerning the testicular toxicity is the germinal epithelium’s loosening [38, 39]. Therefore, quantitative assessment of germinal epithelium area or its diameter has been performed in different studies [1, 30, 31, 36]. It is well-demonstrated that CIS affects testicular germinal epithelium by both cytotoxic effect and induction of apoptosis [40]. The results indicate that CIS administration did not significantly affect the seminiferous area, but it caused a significant decrease in the germinal layer area. Germinal epithelial atrophy is generally accompanied by seminiferous tubule degeneration. Besides, it is observed in the early stage of testicular damage; whereas, seminiferous atrophy is considered an irreversible and end-stage change of testicular damage [41]. The maximum CR dose (100 mg/kg) could prevent germinal epithelium loss in CIS-treated rats in the present study. To our knowledge, no study has been yet investigated the impact of CR treatment on testicular morphology in CIS-induced reproductive damaged animals. However, there are studies in which CR has been investigated on other testicular damage. In the Bayatpour and colleagues’ study, diabetic rats were treated with saline or CR (20 mg/kg i.p.) for 60 days. They reported that untreated diabetic rats exhibited significant testicular damage, but the CR treatment caused a significant prevention in germinal epithelium sloughing [42]. Contrary to Bayatpour and coworkers’ study [42], we found that CR cannot influence germinal layer loss in CIS-treated rats at doses up to 25 mg/kg. It is important to highlight that in the mentioned study, CR was administrated for 60 days while only 13 injections of CR were performed in the current study. In line with germinal epithelium damage, spermatogenesis evaluated using the JS system was significantly decreased in CIS and CIS + CR6.25 and CIS + CR25 groups compared to the control group. CR treatment significantly increased the JS compared to the CIS group only at 100 mg/kg. A recent study demonstrated that 60 days of CR administration (20 mg/kg i.p.) to Wistar rats that simultaneously received high-fat diet plus streptozotocin could effectively prevent spermatogenesis impairment by increasing the JS [43].

Mechanistic studies demonstrated that impairment in oxidant-antioxidant balance and lipid peroxidation play a pivotal role in CIS-induced testicular damage [44]. Another promising finding was that CR could effectively attenuate testicular MDA levels as well as enhance SOD activity in CIS-treated rats at 25 mg/kg and 100 mg/kg doses. This result is in line with earlier studies wherein testicular lipid peroxidation has been decreased with CR treatments [34, 45].

## Conclusions

In conclusion, the present study’s findings demonstrated that CR partially attenuated some of the CIS-related pathological effects on testicular tissue because of its antioxidant property. Numerous studies have shown that CR has anti-cancer efficacy on a wide range of cancer cells. Moreover, CR not only does not interfere with cisplatin but also has synergistic effects. Some studies have also reported the protective effects of crocin on renal, hepatic, and gastrointestinal side effects of chemotherapy drugs. Therefore, it is suggested that CR might be used as a potent therapeutic agent combined with CIS to reduce testicular damage caused by CIS.

## Data Availability

The datasets used in the current study are available from the corresponding author on reasonable request.

## Abbreviations

CIS: Cisplatin
CR: Crocin
PBS: Phosphate buffered saline
TBARS: Thiobarbituric acid reactive species
MDA: Malondialdehyde
SOD: Superoxide dismutase
JS: Johnsen’s scoring
